# Estimated glomerular filtration rate is a biomarker of cognitive impairment in Parkinson’s disease

**DOI:** 10.3389/fnagi.2023.1130833

**Published:** 2023-05-22

**Authors:** Yi Qu, Qi-Xiong Qin, Dan-Lei Wang, Jiang-Ting Li, Jing-Wei Zhao, Ke An, Jing-Yi Li, Zhi-Juan Mao, Zhe Min, Yong-Jie Xiong, Zheng Xue

**Affiliations:** Department of Neurology, Tongji Hospital, Tongji Medical College, Huazhong University of Science and Technology, Wuhan, China

**Keywords:** Parkinson’s disease, kidney function, cognitive function, estimated glomerular filtration rate, biomarkers

## Abstract

**Backgrounds:**

The relationship between kidney function and cognitive impairment in Parkinson’s disease (PD) is poorly understood and underexplored. This study aims to explore whether renal indices can serve as indicators to monitor the cognitive impairment of PD.

**Methods:**

A total of 508 PD patients and 168 healthy controls from the Parkinson’s Progression Markers Initiative (PPMI) were recruited, and 486 (95.7%) PD patients underwent longitudinal measurements. The renal indicators including serum creatinine (Scr), uric acid (UA), and urea nitrogen, as well as UA/Scr ratio and estimated glomerular filtration rate (eGFR), were measured. Cross-sectional and longitudinal associations between kidney function and cognitive impairment were evaluated using multivariable-adjusted models.

**Results:**

eGFR was associated with lower levels of cerebrospinal fluid (CSF) A*β*_1–42_ (*p* = 0.0156) and α-synuclein (*p* = 0.0151) and higher serum NfL (*p* = 0.0215) in PD patients at baseline. Longitudinal results showed that decreased eGFR predicted a higher risk of cognitive impairment (HR = 0.7382, 95% CI = 0.6329–0.8610). Additionally, eGFR decline was significantly associated with higher rates of increase in CSF T-tau (*p* = 0.0096), P-tau (*p* = 0.0250), and serum NfL (*p* = 0.0189), as well as global cognition and various cognitive domains (*p* < 0.0500). The reduced UA/Scr ratio was also linked to higher NfL levels (*p* = 0.0282) and greater accumulation of T-tau (*p* = 0.0282) and P-tau (*p* = 0.0317). However, no significant associations were found between other renal indices and cognition.

**Conclusion:**

eGFR is altered in PD subjects with cognitive impairment, and predict larger progression of cognitive decline. It may assist identifying patients with PD at risk of rapid cognitive decline and have the potential to monitoring responses to therapy in future clinical practice.

## 1. Introduction

Parkinson’s disease (PD) is the second most common neurodegenerative disease affecting both dopaminergic-mediated motor and non-dopaminergic-mediated non-motor systems (Yoo et al., 2019). Cognitive impairment is one of the most prevalent non-motor symptoms especially in the late stages of PD, which involves impairments in specific domains such as visuospatial, attentional, executive, and memory functions (Weintraub et al., 2015). The cognitive decline will reduce treatment compliance, increase extra nursing time, and worsen the quality and prognosis of life for PD patients (Leroi et al., 2012). Moreover, previous research has suggested that PD dementia and Alzheimer’s disease (AD) may share parallel changes in both symptoms and protein profiles (Gomperts et al., 2013), indicating that AD-related proteins have significant roles in PD-related cognitive decline.

Chronic kidney disease (CKD) is characterized by impaired renal function, which is associated with accelerated cognitive decline (Helmer et al., 2011). The renal indices include serum creatinine (Scr), uric acid (UA), and blood urea nitrogen (BUN), among which estimated glomerular filtration rates (eGFR) based on Scr levels are the most widely used to assess renal function, with diagnostic thresholds of less than 60 mL/min/1.73m^2^ for eGFR. Previous studies have reported that the cognitive-related pathological changes in the brain are exacerbated by reduced renal function in those with cognitive impairment (Lopez et al., 2018; Petersen et al., 2019). However, the pathophysiological mechanism linking cognition and kidney function in PD patients remains unclear.

The commonly used tools to detect cognitive impairment include neuropsychological tests, neuroimaging examinations, and fluid biomarkers. Blood biomarkers have the advantages of objectivity and accessibility compared to others and are more suitable for the early identification of cognitive impairment. Likewise, it lacks and urgently needs valid intervention for PD comorbid with cognitive decline. Therefore, this study aims to investigate whether there is an association between eGFR and cognitive function in PD patients from the open-source database Parkinson’s Progression Marker Initiative (PPMI). We hypothesize that the reduced eGFR, which reflects impaired kidney function, would correlate with cognitive decline in PD and can be used as a potential predictive and prognostic tool to evaluate PD progression in clinical practice.

## 2. Methods

### 2.1. Study participants

Data used in this article were obtained from PPMI.^1^ The PPMI is an ongoing, observational, prospective, multicenter study that aims to identify biomarkers for PD deterioration. Participants were included if they were diagnosed with PD at ≥30 years; had two symptoms among bradykinesia, resting tremor, and rigidity, or only asymmetric resting tremor or bradykinesia; had a 2-year period after diagnosis; were not treated for PD; were cognitively normal; and had no potential drug interference. The patients suspected of having progressive supranuclear palsy or multiple system atrophy were excluded from follow-ups to prevent misdiagnosis. Healthy controls (HC) were included if they had no obvious neurologic impairment, they had no family history of PD, and their Montreal Cognitive Assessment (MoCA) score was higher than 26. The subjects in the current study met the criteria of PPMI and were additionally restricted by the age range of 40–85 years and the absence of a medical history of kidney disease (e.g., chronic kidney disease, renal failure, kidney tumors, kidney infection, and kidney resection). Written informed consent was obtained from all recruited participants.

### 2.2. Biomarker analyses

#### 2.2.1. Creatinine determination and eGFR

Biochemical analyses of Scr, UA, and BUN were carried out in Covance laboratories in a uniform fashion according to the study protocol. The eGFR was further calculated based on the levels of Scr using the Chronic Kidney Disease Epidemiology Collaboration (CKD-EPI) equation (Levey et al., 2009). Besides, patients whose eGFR was <60 mL/min/1.73m^2^ were considered to have significant renal function insufficiency (Sunder-Plassmann and Hörl, 2004).

#### 2.2.2. CSF and serum biomarkers

Blood and CSF sample were well collected and performed according to the PPMI biologics manual (Kang et al., 2013). The levels of CSF A*β*_1–42_, Total-tau (T-tau), and phosphorylated-tau 181 (P-tau) were measured using Elecsys® electrochemiluminescence immunoassays on the cobas e 601 analysis platform (Roche Diagnostics; Shaw et al., 2018). CSF total α-synuclein (α-syn) was detected using BioLegend (San Diego, CA) by means of sandwich immunoassay (Kang et al., 2016), while serum NfL was measured with SIMOA^®^ HD-1 analyzer (Quanterix, Lexington, MA, United States; Sampedro et al., 2020). All subjects had planned follow-up with blood collection at 3-month intervals during year one followed by 6-month intervals and CSF collection at 6- and 12-month visits followed by 12-month intervals (Marek et al., 2018).

### 2.3. Cognitive assessments

The cognitive levels were assessed by MoCA. Cognitive indicators for several specific areas that were corrected by published norms, included verbal episodic memory (Hopkins Verbal Learning Test [HVLT] Immediate Recall; HVLT Delayed Recall; HVLT Recognition), visuospatial ability (Judgment of Line Orientation [JoLO]), executive function/working memory (Letter Number Sequencing [LNS]), language (Semantic Fluency Test), and processing speed/attention (Symbol Digit Modality Test [SDMT]) (Kang et al., 2013). Cognitive status was defined as cognitively normal (MoCA>26), cognitive impairment (MoCA 26–22), and dementia (MoCA<22; Litvan et al., 2012). Cognitive function was assessed more than once for each patient. The cognitive decline during follow-ups was defined as scoring at least two cognitive tests that are more than 1.5 standard deviations below normal at baseline and having no functional impairment due to cognitive impairment, which was consistent with the previous description (Ray et al., 2018). The interval between the two assessments was approximately half a year in the first 5 years and 1 year in the following years.

### 2.4. Covariables

Demographic information such as age, sex, years of education, disease duration, and *Apolipoprotein E* (*APOE*) *ε4* status was previously reported to be associated with cognitive decline in PD. Moreover, the assessment of PD symptoms such as disease severity and motor function were recorded by Hoehn-Yahr stages and the Movement Disorders Society Unified Parkinson’s Disease Rating Scale (MDS-UPDRS) III.

### 2.5. Statistical analyses

Differences in demographic characteristics between PD and HC were assessed using the Mann–Whitney U test and *χ*^2^ test. The concentrations of fluid biomarkers did not confirm normal distribution (Kolmogorov–Smirnov test, *p* < 0.0500) and were log10-transformed to achieve normality using the “car” package. Baseline associations between renal and cognitive indicators were explored by multiple linear regression (MLR) models that adjusted for age, sex, educational levels, *APOE ε4* status, and disease duration. Kaplan–Meier curves were then conducted to compare the cumulative probability risk of cognitive impairment during follow-up among different eGFR tertile groups, while multivariate Cox regression models were employed to analyze the association between continuous baseline renal indicators and cognitive progression. The Cox regression was checked by using Schoenfeld residuals and log-minus-log plots. Furthermore, linear mixed-effects (LME) models were used to explore kidney function and the rate of change in cognitive indicators. For sensitivity analyses, we performed subgroup analyses by age, sex, and *APOE ε4* status to explore the possible effect modification of these factors on the associations between renal and cognitive function by adding the interaction term to the MLR and LME models.

All statistical analyses were conducted using R software version 4.1.3, and the statistical significance threshold was set at a two-tailed *p* < 0.05.

## 3. Results

### 3.1. Study participants

**Figure 1:**
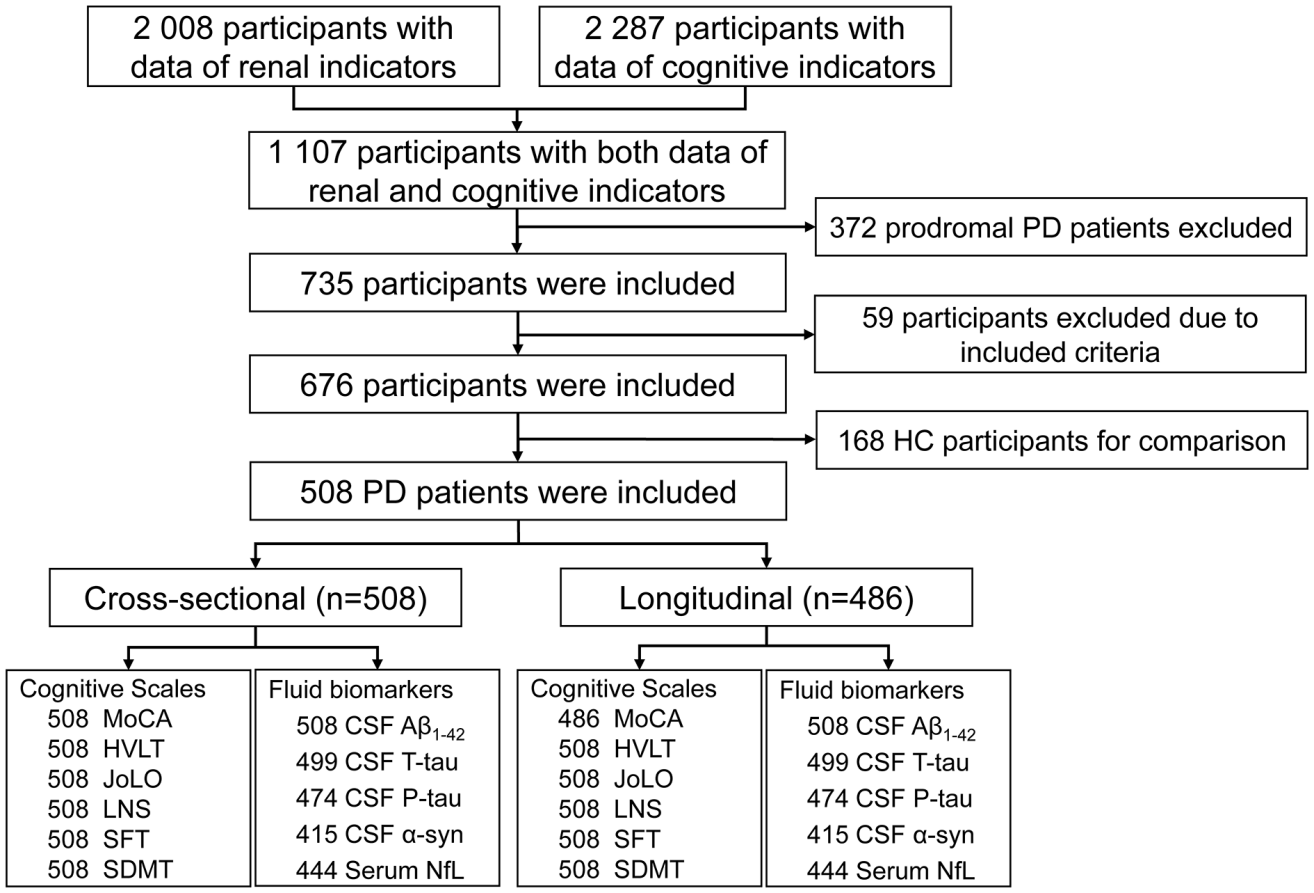
Flowchart of data analysis. A*β*, amyloid beta; CSF, cerebrospinal fluid; HC, health control; HVLT, Hopkins verbal learning test; JoLO, judgment of line orientation; LNS; letter number sequencing; M, male; MDS-UPDRS, Movement disorders unified Parkinson’s disease rating scale; MoCA, Montreal cognitive assessment; NfL, neurofilament light chain; PD, Parkinson’s disease; SDMT, symbol digit modality test; SFT, semantic fluency test.

The flow chart and demographic information of the study participants are listed in Figure 1 and Table 1. Briefly, 508 clinically defined PD patients and 168 HC subjects were included. As expected, the performances on the cognitive assessment differed between both groups, and PD patients had lower levels of CSF A*β*_1-42_, T-tau, P-tau, and α-syn, as well as higher serum NfL. There was no difference in age, sex, or *APOE ε4* status. Finally, 486 *de novo* PD patients were involved in longitudinal analyses. During the up-to-ten-year follow-ups, 184 individuals (39.5%) of 466 non-dementia PD patients had a cognitive decline, and 82 (17.6%) converted to dementia.

**Table 1:** Baseline characteristics of included participants.

Characteristics	HC (*n* = 168)	Patients with PD (*n* = 508)	*p*-value
Age (y)	61.7 (9.7)	62.1 (8.5)	0.648
Gender (F/M)	62/106	191/317	0.872
Education (y)	15.9 (2.9)	15.5 (3.5)	0.317
*APOE ε4* carriers (%)	44 (26.2)	122 (24.0)	0.555
Disease duration (y)	–	2.7 (2.8)	–
H-Y stage	–	1.6 (0.5)	–
UPDRS-III	–	20.9 (9.7)	–
Scr (μmol/L)	85.2 (16.5)	83.7 (17.0)	0.313
UA (μmol/L)	319.8 (7.1)	312.2 (78.3)	0.174
BUN (mmol/L)	6.0 (1.5)	6.2 (1.6)	0.286
eGFR (ml/min/1.73m^2^)	78.4 (14.1)	79.3 (14.0)	0.344
CSF Aβ42 (pg/mL)	998.9 (421.0)	871.7 (344.0)	**0.001**
CSF T-tau (pg/mL)	184.5 (62.5) (*n* = 166)	163.9 (52.5) (*n* = 499)	**<0.001**
CSF P-tau (pg/mL)	16.6 (5.9) (*n* = 156)	14.2 (4.7) (*n* = 474)	**<0.001**
CSF *α*-syn (pg/mL)	1640.9 (610.2) (*n* = 168)	1433.3 (546.4) (*n* = 415)	**<0.001**
Serum NfL (pg/mL)	11.5 (5.3) (*n* = 152)	13.0 (5.9) (*n* = 444)	**0.004**
MoCA	28.2 (1.1)	26.8 (2.6)	**<0.001**
HVLT total recall	49.2 (10.2)	45.7 (10.9)	**<0.001**
HVLT delayed recall	48.7 (11.1)	44.8 (11.5)	**<0.001**
HVLT recognition	47.9(11.7)	44.9 (11.2)	**<0.001**
JoLO	12.3 (2.9)	11.7 (3.0)	**0.017**
LNS	11.7 (2.7)	11.3 (2.8)	0.121
Semantic fluency test	53.1 (10.7)	50.9 (10.2)	0.076
SDMT	50.3 (10.3)	44.8 (9.8)	**<0.001**

A*β*, amyloid beta; APOE, Apolipoprotein E; *α*-syn, *α*-synuclein; BUN, blood urea nitrogen; CSF, cerebrospinal fluid; eGFR, estimated glomerular filtration rate; F, female; HC, healthy control; HVLT, Hopkins verbal learning test; JoLO, judgment of line orientation; LNS; letter number sequencing; M, male; MDS-UPDRS, Movement disorders unified Parkinson’s disease rating scale; MoCA, Montreal cognitive assessment; n, number; NfL, neurofilament light chain; PD, Parkinson’s disease; Scr, serum creatinine; SDMT, symbol digit modality test; UA, uric acid; y, year. The bold values were symbolized to statistically significant difference (*p* < 0.05).

### 3.2. Cross-sectional analyses and subgroup analyses between renal indicators and cognition

**Figure 2:**
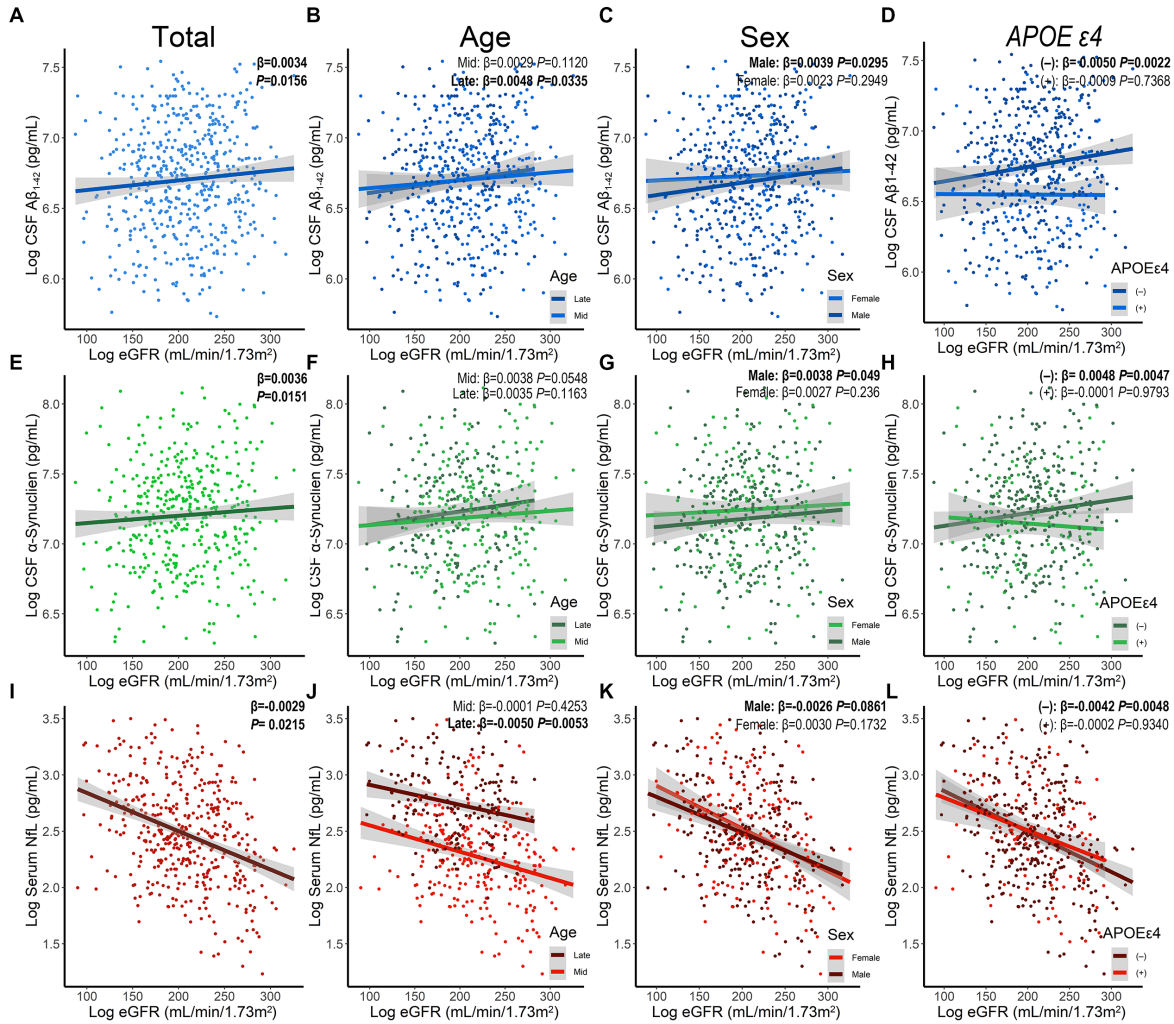
The association between baseline eGFR and levels of CSF Aβ_1-42_ **(A–D)**, CSF *α*-synuclein **(E–H)**, and serum NfL **(I–L)** stratified by age, sex, and *APOE ε4* status. A*β*, amyloid beta; *α*-syn, *α*-synuclein; *APOE, Apolipoprotein E*; CSF, cerebrospinal fluid; eGFR, estimated glomerular filtration rate; NfL, neurofilament light chain; PD, Parkinson’s disease.

Using MLR models after adjusting for age, sex, educational levels, *APOE ε4* status, and disease duration, results showed that decreased eGFR was associated with reduced levels of CSF A*β*_1–42_ (*β* = 0.0034, *p* = 0.0156) and *α*-syn (*β* = 0.0036, *p* = 0.0151), as well as increased concentration of serum NfL (*β* = −0.0029, *p* = 0.0215; Figure 2; Table 2). However, no significant associations were found between baseline eGFR and cognitive scales. The levels of Scr were not correlated with CSF A*β*_1–42_ and *α*-syn, while UA and BUN were not associated with any cognitive indicators. Additionally, the lower UA/Scr ratio was linked to a higher concentration of serum NfL (*β* = −0.1561, *p* = 0.0282), indicating that the reduced UA and increased Scr levels were observed in PD patients with worse cognition. Considering the potential influence of covariables, the interaction and subgroup analyses were conducted to discover the detailed results in different groups. We found that these associations remained significant in late-age (≥65 years), male, and *APOE ε4* (−) participants (Supplementary Tables S1–S4).

**Table 2:** Baseline associations between renal and cognitive indicators in patients with *de novo* PD.

Cognitive indicators	Scr		UA		BUN		UA/Scr		eGFR	
	*β*	*p*-value	*β*	*p*-value	*β*	*p*-value	*β*	*p*-value	*β*	*p*-value
CSF A*β*42	**−0.0033**	**0.0094**	−0.0017	0.7243	0.0407	0.5959	0.1275	0.1050	**0.0034**	**0.0156**
CSF T-tau (*n* = 499)	−0.0002	0.0626	0.0001	0.7072	−0.0020	0.6723	0.0091	0.0693	0.0015	0.1680
CSF P-tau (*n* = 474)	−0.0004	0.1990	0.0005	0.6470	0.0130	0.4030	0.0218	0.1810	0.0004	0.1410
CSF α-syn (*n* = 415)	**−0.0036**	**0.0096**	0.0004	0.9428	0.1047	0.1842	0.1613	0.0519	**0.0036**	**0.0151**
Serum NfL (*n* = 444)	0.0022	0.0696	−0.0037	0.4029	0.0815	0.2297	**−0.1561**	**0.0282**	**−0.0029**	**0.0215**
MoCA	0.0002	0.0938	−0.0001	0.7881	−0.0115	0.1674	−0.0151	0.0730	−4986.0	0.2505
HVLT total recall	−0.0003	0.4320	0.0001	0.9584	−0.0279	0.1697	0.0070	0.7360	0.0302	0.3947
HVLT delayed recall	−0.0002	0.5723	−0.0006	0.6570	−0.0285	0.2124	−0.0095	0.6857	0.2097	0.4377
HVLT recognition	−0.0005	0.2090	−0.0020	0.1880	−0.0038	0.8759	−0.0081	0.7416	0.6959	0.6579
JoLO	−0.0001	0.8936	−0.0025	0.1097	−0.0283	0.2402	−0.0313	0.2004	−0.1606	0.1410
LNS	0.0007	0.0592	0.0018	0.2240	0.0208	0.3660	−0.0054	0.8190	−0.0166	0.0698
Semantic fluency test	0.0004	0.2565	−0.0009	0.4277	−0.0016	0.9302	−0.0334	0.0775	−0.0416	0.2424
SDMT	0.0005	0.1156	0.0003	0.8101	0.0045	0.8191	−0.0336	0.0929	−0.0549	0.1004

The models were adjusted for age, sex, educational levels, APOE *ε*4 status, and disease duration.

A*β*, amyloid beta; APOE, Apolipoprotein E; *α*-syn, α-synuclein; BUN, blood urea nitrogen; CSF, cerebrospinal fluid; eGFR, estimated glomerular filtration rate; HVLT, Hopkins verbal learning test; JoLO, judgment of line orientation; LNS; letter number sequencing; MoCA, Montreal cognitive assessment; n, number; NfL, neurofilament light chain; PD, Parkinson’s disease; Scr, serum creatinine; SDMT, symbol digit modality test; UA, uric acid. The bold values were symbolized to statistically significant difference (*p* < 0.05).

### 3.3. Longitudinal analyses and subgroup analyses between renal indicators and cognition

We performed longitudinal analyses to examine the predictive value of kidney function on cognitive changes for patients with PD. In short, the incidence rate of cognitive impairment or dementia progression was 211.3 per 1,000 patients per year for those with eGFR <60 mL/min/1.73m^2^, compared to 85.8 per 1,000 patients per year for those with eGFR between 60 and 90 mL/min/1.73m^2^, and 45.8 per 1,000 patients per year for those with eGFR >90 mL/min/1.73m^2^.

**Figure 3:**
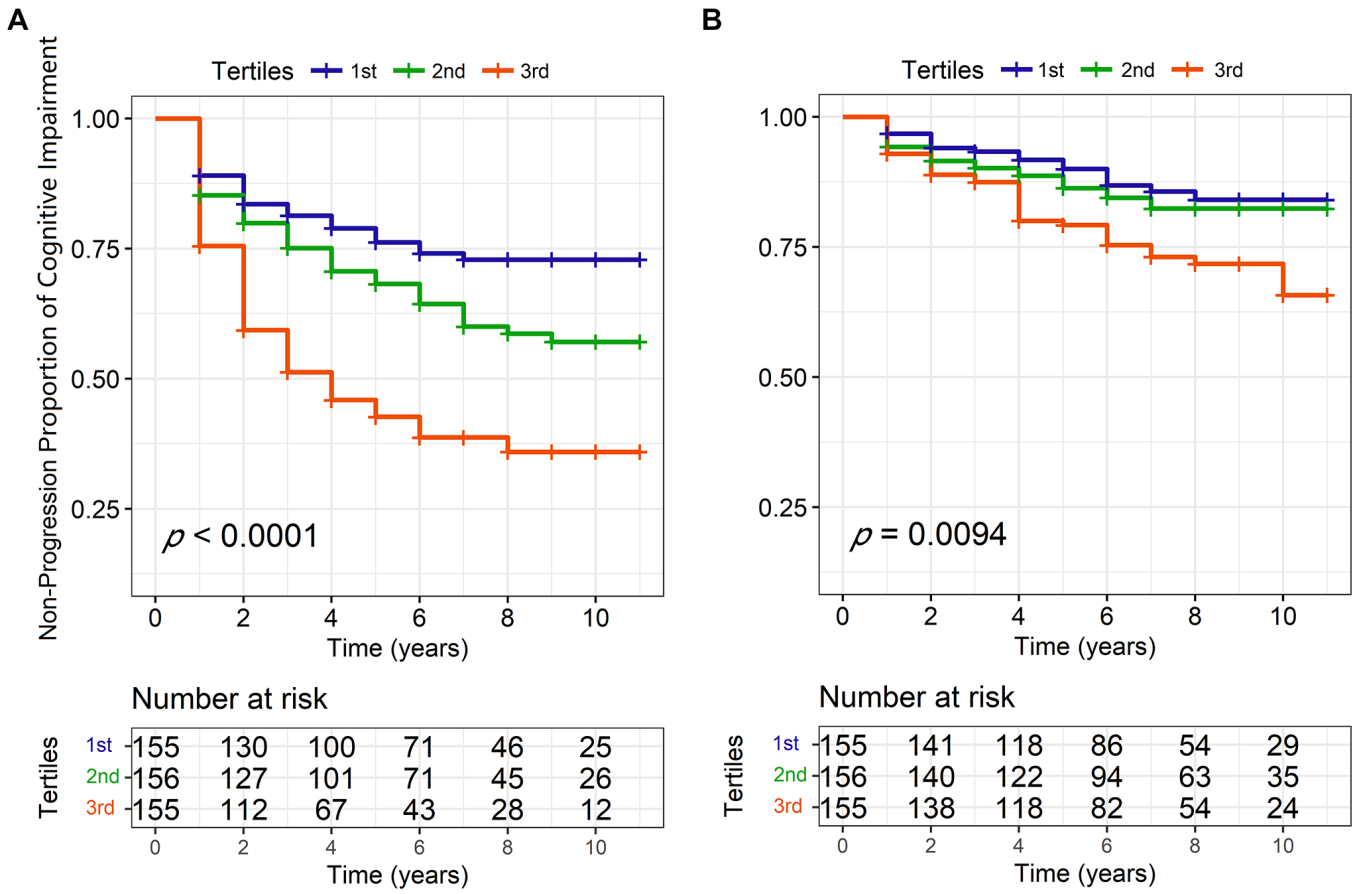
Kaplan–Meier curves of eGFR tertiles for conversion to cognitive impairment **(A)** and dementia **(B)** during 10-year follow-ups. eGFR, estimated glomerular filtration rate; PD, Parkinson’s disease.

On the one hand, we found that lower baseline eGFR might predict the higher risk of cognitive impairment progression using Cox models after IPW (hazard ratio [HR] = 0.7382, 95% confidence interval [CI] = 0.6329–0.8610, *p* = 0.0001). The Kaplan–Meier analysis and log-rank test also showed significant differences among the tertiles of eGFR (Log-rank *p* < 0.0001, Figure 3A). However, we did not see a correlation between baseline eGFR and the risk of dementia in the Cox models (HR = 0.8823, 95%CI = 0.7036–1.106, *p* = 0.2780) although the Kaplan–Meier analysis showed some discrepancies among the tertiles (Log-rank *p* = 0.0094, Figure 3B). Moreover, renal function insufficiency (eGFR <60 ml/min/1.73m^2^) was proven to be associated with a higher risk of cognitive impairment (HR = 2.0936, 95% CI = 1.2026–3.6449, *p* = 0.0090; Figure 1) but not with the risk of dementia (HR = 1.0756, 95% CI = 0.4651–2.4870, *p* = 0.8647). On the other hand, decreased baseline eGFR was significantly associated with a higher increased rate of CSF T-tau (*β* = −0.00008, *p* = 0.0096), CSF P-tau (*β* = −0.00006, *p* = 0.0250), and serum NfL (*β* = −0.00047, *p* = 0.0189; Table 3) using LME models. The reduced UA/Scr ratio also predicted greater accumulation of T-tau (*β* = −0.00110, *p* = 0.0282) and P-tau (*β* = −0.00087, *p* = 0.0317). Additionally, lower baseline eGFR predicted greater decline not only in global cognitive levels (MoCA, β = 474.600, *p* = 0.0070), verbal episodic memory (HVLT Total Recall, *β* = 0.03093, *p* = 0.0311; HVLT Delayed Recall, β = 0.14571, *p* = 0.0146), language (Semantic Fluency Test, *β* = 0.02965, *p* = 0.0044), and processing speed/attention (SDMT, *β* = 0.01398, *p* = 0.001). Furthermore, increased Scr concentrations at baseline indicated greater deterioration of serum NfL (*β* = 0.0038, *p* = 0.0230) and larger decline scores of the Semantic Fluency Test (*β* = −0.01966, *p* = 0.0236). Higher UA levels predicted more severe progression of executive function (LNS, *β* = −0.00137, *p* = 0.0201), while elevated BUN was associated with lower scores of LNS (*β* = −0.08129, *p* = 0.0034) and SDMT (*β* = −0.10067, *p* = 0.0500).

**Table 3:** Longitudinal associations between renal and cognitive indicators in patients with *de novo* PD.

Cognitive indicators	Scr		UA		BUN		UA/Scr		eGFR	
Cox models	OR	95% CI	OR	95% CI	OR	95% CI	OR	95% CI	OR	95% CI
Cognitive impairment and dementia	**1.3488**	**1.1553–1.5748**	1.0714	0.8986–1.2770	1.0458	0.9519–1.1489	**0.8248**	**0.6842–0.9944**	**0.7382**	**0.6329–0.8610**
Dementia	1.1449	0.9164–1.4300	1.1909	0.9216–1.5390	1.0239	0.8889–1.1790	1.0537	0.8109–1.3690	0.8823	0.7036–1.1060
LME models	*β*	*p*-value	*β*	*p*-value	*β*	*p*-value	*β*	*p*-value	*β*	*p*-value
CSF Aβ42 (*n* = 426)	−0.00023	0.8877	−0.00044	0.2012	−0.01273	0.4460	−0.05168	0.1134	−0.00158	0.4258
CSF T-tau (*n* = 418)	−0.00003	0.2692	−0.00001	0.1230	−0.00001	0.9840	**−0.00110**	**0.0282**	**−0.00008**	**0.0096**
CSF P-tau (*n* = 395)	0.00002	0.2785	−0.00001	0.1312	−0.00001	0.9852	**−0.00087**	**0.0317**	**−0.00006**	**0.0250**
CSF *α*-syn (*n* = 341)	0.00259	0.1159	−0.00044	0.2150	0.01487	0.3710	−0.00048	0.9886	−0.00313	0.1097
Serum NfL (*n* = 442)	**0.00038**	**0.0230**	0.00004	0.2802	0.00135	0.4199	−0.00262	0.4496	**−0.00047**	**0.0189**
MoCA	−127.9	0.3759	5.17100	0.8660	−2257.4	0.1231	2026.00	0.4800	**474.600**	**0.0070**
HVLT Total Recall	−0.01597	0.1734	−0.00341	0.1675	−0.16080	0.1626	−0.16903	0.4631	**0.03093**	**0.0311**
HVLT Delayed Recall	−0.04033	0.4203	−0.00200	0.8478	−0.78980	0.1089	0.19202	0.8455	**0.14571**	**0.0146**
HVLT Recognition	−0.39810	0.7588	0.24020	0.3807	−23.100	0.0694	25.0470	0.3250	1.97700	0.2020
JoLO	−0.01412	0.1226	−0.00283	0.1410	0.01844	0.8390	−0.04316	0.8091	0.00817	0.4601
LNS	−0.00420	0.1330	**−0.00137**	**0.0201**	**−0.08129**	**0.0034**	−0.08510	0.1226	0.00297	0.4152
Semantic Fluency Test	**−0.01966**	**0.0236**	−0.00304	0.0993	−0.14061	0.1048	0.02960	0.8632	**0.02965**	**0.0044**
SDMT	−0.00605	0.2356	−0.00170	0.1140	**−0.10067**	**0.0500**	−0.12530	0.2110	**0.01398**	**0.0229**

The models were adjusted for age, sex, educational levels, APOE ε4 status, and disease duration.

A*β*, amyloid beta; APOE, Apolipoprotein E; *α*-syn, *α*-synuclein; BUN, blood urea nitrogen; CI, confidence intervals; CSF, cerebrospinal fluid; eGFR, estimated glomerular filtration rate; HVLT, Hopkins verbal learning test; JoLO, judgment of line orientation; LME, linear mixed-effects; LNS; letter number sequencing; MoCA, Montreal cognitive assessment; *n*, number; NfL, neurofilament light chain; OR, odd ratio; PD, Parkinson’s disease; Scr, serum creatinine; SDMT, symbol digit modality test; UA, uric acid. The bold values were symbolized to statistically significant difference (*p* < 0.05).

We further examined these relationships among PD patients stratified by age, sex, and *APOE ε4* status (Supplementary Tables S5–S7). Consistent with cross-sectional results, significant differences were observed in late-age and male patients. Unlike the cross-sectional results, lower eGFR levels represented more decline risk of verbal episodic memory (HVLT Total Recall, *β* = 0.07365, *p* = 0.0121; HVLT Delayed Recall, *β* = 0.27800, *p* = 0.0341; HVLT Recognition, *β* = 8.13600, *p* = 0.0341, *p* = 0.0163; Supplementary Table S7) in *APOE ε4* (+) carriers.

## 4. Discussion

This prospective study demonstrated significant associations between impaired kidney function and global cognitive impairment in patients with *de novo* PD independent of confounding variables, as well as specific cognitive domains such as episodic memory, language, processing speed, and attention. Remarkably, decreased eGFR was related to lower levels of CSF A*β*_1-42_ and *α*-syn and higher levels of serum NfL at baseline. Meanwhile, decreased eGFR predicted a greater accumulation of CSF T-tau, P-tau, and serum NfL in up-to-seven-year follow-ups. These results suggested that eGFR might be a potential blood biomarker for predicting and monitoring cognitive changes in patients with *de novo* PD compared with other renal indicators.

The relationship of kidney function with subsequent cognitive decline in early PD patients remains unclear in previous studies although it has been studied that poor performances of kidney function are associated with an increased risk of cognitive impairment in the elderly population (Miyazawa et al., 2018; Fujiyoshi et al., 2020). These associations may be related to atherosclerosis, cerebral small vessel diseases, hematopoietic function impairment, or methylation metabolism that are secondary to CKD. Impaired kidney function is a very common condition in the general population, which can be easily monitored using blood tests. In this context, GFR, calculated by Scr, is useful in reflecting glomerular function and employed to manage CKD over the past two decades (Levey et al., 2007). There is a long-term process for eGFR affecting cognition. Previous studies found that reduced eGFR was linked to cognitive impairment in the population with normal renal function (Davey et al., 2013), and the one unit decrease of eGFR per year indicated a higher risk of cognitive decline (Barzilay et al., 2013). Additionally, another study showed that elevated Scr levels were correlated with the severity and duration of PD (Zhong et al., 2018), and patients with CKD had an increased risk of PD, suggesting a common pathophysiological process between CKD and PD (Nam et al., 2019). This might be because the decreased eGFR could lead to toxic accumulation, resulting in neurotoxic effects or neuroinflammatory reactions (Zhong et al., 2018). In the current study, we found that PD patients had worse cognitive performances; however, no obvious kidney impairment occurred in patients with *de novo* PD. The main reason might be attributed to the short disease duration of the included PD participants.

The exact mechanism underlying the association of kidney function with subsequent cognitive impairment in PD is unknown but some plausible explanations have been suggested. We observed lower levels of CSF Aβ_1-42_, T-tau, and P-tau, as well as higher serum NfL in patients with PD compared with controls. Amyloid plaques and neurofibrillary tangles coexist in PD brains (Jellinger, 2008), and both amyloid and tau pathology can dynamically interact with the *α*-synuclein misfolding process according to previous studies (Irwin et al., 2017), which may explain our findings. Nevertheless, controversies still persist about the relationships between fluid biomarkers and PD (Parnetti et al., 2019). AD pathology may also be an important co-pathology and driver of adverse disease outcomes. To the best of our knowledge, this is the first time there is a report on the associations between eGFR and AD-related biomarkers in patients with PD. The results indicated that PD patients with worse kidney function had lower levels of CSF Aβ_1-42_ and α-syn, as well as higher levels of serum NfL at baseline, while they had greater accumulation of CSF T-tau, P-tau, and serum NfL at 7-year follow-up. Some scholars argue that the correlation between AD pathology and renal function can be explained by tau protein being transported from the brain to the peripheral blood and cleared in the liver and kidney (Liu et al., 2015). Another study also proposes that the increased clearance rate of peripheral tau will reduce tau accumulation and neurodegeneration in the brain, which may be a potential treatment method for PD (Shi et al., 2016). Alternatively, an impaired renal function could accelerate the pathological process of Aβ and tau. CKD can cause oxidative stress and hyperhomocysteinemia via vascular injury and endothelial dysfunction (Etgen et al., 2012; An et al., 2017). Abnormal levels of serum homocysteine are involved in the pathology of cognitive impairment as they may enhance A*β* production and mediate its neurotoxicity (Vinothkumar et al., 2017). These factors may jointly contribute to the increased risk of cognitive impairment in PD. Furthermore, severe CKD and uremia may cause metabolic derangements in the basal ganglia, and inadequate dialysis has been reported to result in basal ganglia injury (Cupidi et al., 2006). Patients with CKD are particularly susceptible to aluminum and manganese toxicity (Ohtake et al., 2005). Therefore, PD-related exacerbation caused by CKD can aggravate cognitive impairment through disease progression. However, our findings did not show an association between PD and renal indicators, which did not support this hypothesis.

It was noted that the mechanisms of synergetic effect on neurodegenerative and vascular damage in the brain may contribute to cognitive decline in patients with poor kidney function (Guerville et al., 2021). According to neuroimaging results, cognitive impairment associated with lower eGFR is involved in several brain functional domains and is responsible for the damage of multiple cortical regions (especially the frontal lobe) and subcortical modulatory neurons, particularly adrenergic neurons in the mesencephalon and cholinergic neurons in the nucleus basalis of Meynert (Viggiano et al., 2020). Our analyses indicated that worse renal function was associated with poorer performances in both global and specific cognitive domains, which reflect the functional degeneration of different brain regions. Consistent with previous research, these observations suggest that renal impairment in PD is more closely related to multiple dysfunctions among cognitive domains, which mainly involved executive function, delayed recall, language, and attention (Kurella et al., 2005; Lee et al., 2015). In addition, our finding showed that PD patients with higher UA levels had a faster progression of executive function, which was similar to previous results (Vannorsdall et al., 2008; Latourte et al., 2018), whereas those patients with higher BUN levels were also predicted to have a larger decline in language and attention. The mechanism of this relationship with PD is still unclear, and further studies are needed to clarify whether kidney function affects cognitive impairment in patients with PD from the neuroimaging perspective.

The subgroup results indicate that there are significant associations between eGFR and cognitive impairment in older and male populations. We, therefore, propose that elderly people and men with low levels of eGFR have a higher risk of cognitive impairment and cognitive-related pathology accumulation in *de novo* PD and suggest that improving eGFR will provide greater benefits for those populations at high risk of cognitive impairment. Besides, the obvious relationships also emerge in *APOE ε4* negative carriers, implying that the results can be further generalized to the community- and population-based studies.

The main strengths of this study were that both cross-sectional and prospective study designs were adopted, and the associations between eGFR and biomarkers of AD pathology were reported for the first time. However, it also had some limitations that need to be addressed. First, the study only observed a correlation between reduced eGFR and cognitive impairment but did not elucidate the underlying mechanism, which warrants further investigation. Second, the study population consisted mostly of middle-aged adults (50–70 years), whereas dementia and CKD are more prevalent in older age groups. Therefore, future studies should include older participants to examine the association more comprehensively. Third, the study relied on eGFR as the sole indicator of renal function, without considering other measures such as albuminuria and cystatin C, which were not available in our cohort. Additionally, lifestyle factors and cholesterol metabolites may also affect renal function and should be taken into account in future analyses.

## 5. Conclusion

To sum up, this study demonstrated that eGFR levels are reduced in *de novo* PD subjects with cognitive impairment. As well, decreased eGFR and UA/Scr predicted worsening cognitive function over time in PD patients. Notably, there were cross-sectional associations of eGFR with the levels of CSF A*β*_1–42_, *α*-syn, and serum NfL, as well as longitudinal relationships of eGFR with the levels of CSF T-tau, P-tau, and serum NfL. These findings suggest that eGFR and UA/Scr ratio can act as potential diagnostic and prognostic biomarkers of cognitive impairment in *de novo* PD. It can assist clinicians and researchers in identifying patients with PD at risk of rapid cognitive decline and may have the potential for monitoring responses to therapy in future clinical practice.

## Data availability statement

The datasets presented in this study can be found in online repositories. The names of the repository/repositories and accession number(s) can be found in the article/Supplementary material. The clinical data used in this study from the PPMI cohort are available at the PPMI website (https://www.ppmi-info.org/access-data-specimens/download-data/).

## Author contributions

ZX and YQ designed and conceptualized the study. YQ, J-TL, KA, J-YL, Q-XQ, D-LW, and J-WZ conducted the study. YQ, Z-JM, Y-JX, Z-JM, and ZX analyzed and extracted the data. YQ, J-TL, KA, and ZX wrote the first draft of the manuscript. All authors contributed to the article and approved the submitted version.

## Funding

This study was funded by the National Natural Science Foundation of Hubei Province (grant number 2020CFB590) and the Fundamental Research Funds for the Central Universities (YCJJ202201020). PPMI (a public-private partnership) is funded by the Michael J. Fox Foundation for Parkinson’s Research and multiple funding partners.

## Conflict of interest

The authors declare that the research was conducted in the absence of any commercial or financial relationships that could be construed as a potential conflict of interest.

## Publisher’s note

All claims expressed in this article are solely those of the authors and do not necessarily represent those of their affiliated organizations, or those of the publisher, the editors and the reviewers. Any product that may be evaluated in this article, or claim that may be made by its manufacturer, is not guaranteed or endorsed by the publisher.

## Footnotes

^1^ www.ppmi-info.org/data
