# Supplementary material for: Estimated glomerular filtration rate is a biomarker of cognitive impairment in Parkinson’s disease
Source: Front Aging Neurosci. 2023 May 22;15:1130833. doi: 10.3389/fnagi.2023.1130833 (PMC10240071; doi:10.3389/fnagi.2023.1130833)
Supplement: Supplementary file 1 [file Data_Sheet_1.docx]

Supplementary Material

# Supplementary Figures and Tables

## Supplementary Figures


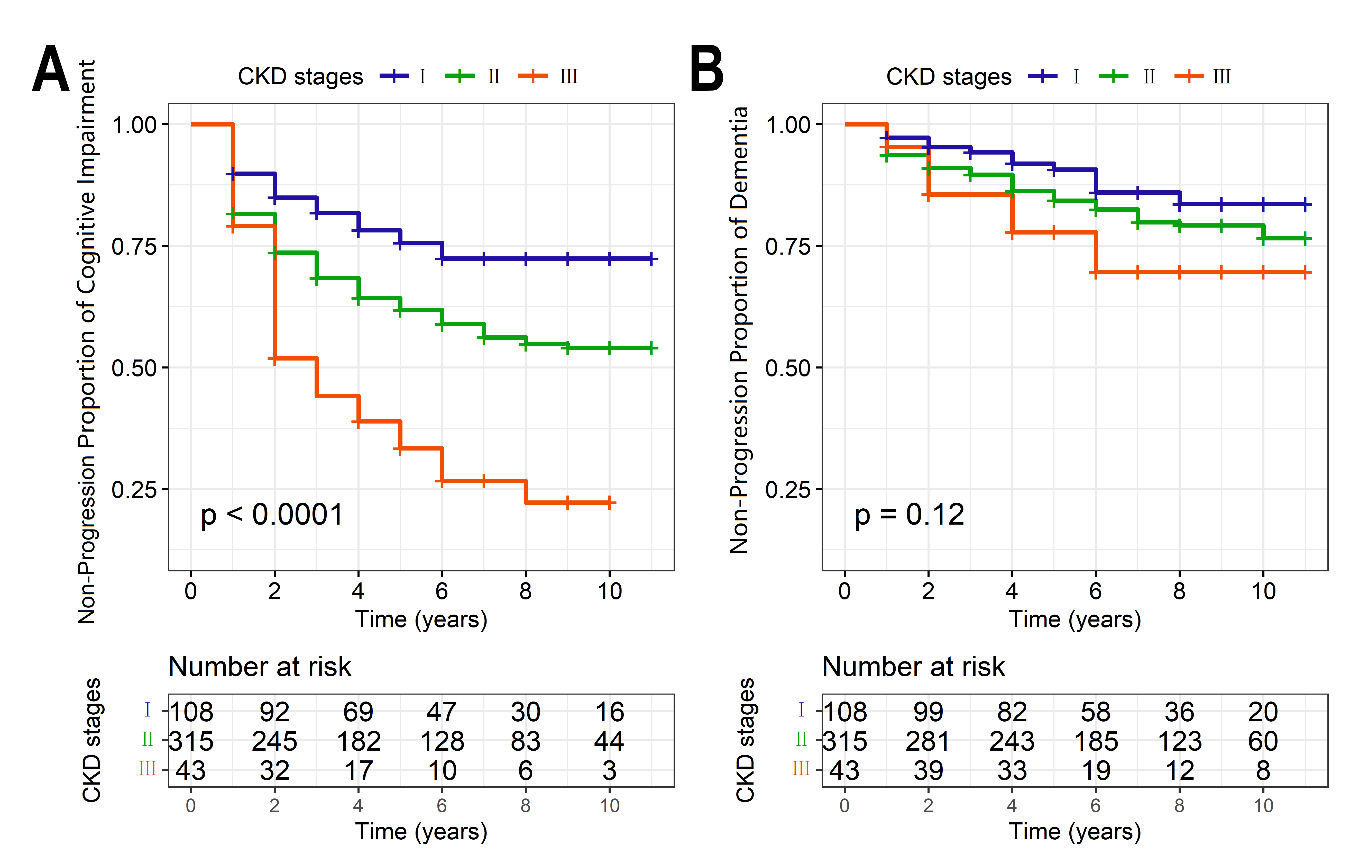


**Supplementary Figure 1. Kaplan-Meier curves of CKD stages for conversion to cognitive impairment (a) and dementia (b) during 10-year follow-ups.**

## Supplementary Tables

**Supplementary Table 1. The interaction analyses of cognition with eGFR and age, sex, and *APOE ε4*.**

| **Cognitive indicators** | Age | | Gender | | *APOE ε4* | |
| --- | --- | --- | --- | --- | --- | --- |
|  | β | P-value | β | P-value | β | P-value |
| CSF Aβ42 | 0.00013 | 0.3940 | 0.00213 | 0.4170 | -0.00340 | 0.1758 |
| CSF T-tau | 0.00001 | 0.2350 | 0.00011 | 0.5302 | -0.00010 | 0.5322 |
| CSF P-tau | -0.00003 | 0.3720 | 0.00078 | 0.1540 | -0.00065 | 0.2138 |
| CSF α-syn | 0.00004 | 0.8163 | 0.00141 | 0.6000 | -0.00448 | 0.0724 |
| Serum NfL | 0.00001 | 0.9315 | 0.00074 | 0.7548 | 0.00199 | 0.3727 |
| MoCA | 0.00001 | 0.4610 | -0.00003 | 0.9226 | 0.00004 | 0.8917 |
| HVLT Total Recall | -0.00002 | 0.5443 | 0.00009 | 0.8922 | 0.00028 | 0.6785 |
| HVLT Delayed Recall | -0.00001 | 0.8720 | -0.00041 | 0.6050 | -0.00026 | 0.7310 |
| HVLT Recognition | 0.00001 | 0.7943 | -0.00013 | 0.8720 | -0.00056 | 0.4780 |
| JoLO | -0.00002 | 0.6760 | -0.00090 | 0.2771 | 0.00046 | 0.5598 |
| LNS | 0.00003 | 0.3950 | 0.001096 | 0.1606 | 0.00016 | 0.8278 |
| Semantic Fluency Test | 0.00007 | 0.0720 | -0.00049 | 0.4450 | 0.00059 | 0.3311 |
| SDMT | 0.00004 | 0.2590 | -0.00075 | 0.2650 | -0.00018 | 0.7742 |

**Supplementary Table 2. Baseline associations between eGFR and cognitive indicators in patients with *de novo* PD stratified by age.**

| **Cognitive indicators** | Mid-age (<65 years) (n=298) | | | | | | Late-age (≥65 years) (n=210) | | | | | |
| --- | --- | --- | --- | --- | --- | --- | --- | --- | --- | --- | --- | --- |
|  | β | SE | F | df | P-value | R^2^ | β | SE | F | df | P-value | R^2^ |
| CSF Aβ42 | 0.0029 | 0.0018 | 2.64 | 291 | 0.1120 | 0.032 | **0.0048** | **0.0023** | **3.31** | **203** | **0.0335** | **0.062** |
| CSF T-tau | 0.0019 | 0.0014 | 1.51 | 285 | 0.1708 | 0.010 | 0.0011 | 0.0017 | 4.11 | 200 | 0.5071 | 0.083 |
| CSF P-tau | 0.0006 | 0.0004 | 1.20 | 268 | 0.1328 | 0.004 | 0.0003 | 0.0005 | 3.69 | 192 | 0.5490 | 0.075 |
| CSF α-syn | 0.0038 | 0.0020 | 2.91 | 247 | 0.0548 | 0.043 | 0.0035 | 0.0022 | 2.46 | 154 | 0.1163 | 0.052 |
| Serum NfL | -0.0010 | 0.0018 | 14.42 | 250 | 0.4253 | 0.239 | **-0.0050** | **0.0017** | **7.56** | **180** | **0.0053** | **0.175** |
| MoCA | -1888.0 | 5764.0 | 5.28 | 291 | 0.7436 | 0.080 | -6926.0 | 6766.0 | 0.78 | 203 | 0.3072 | -0.006 |
| HVLT Total Recall | 0.0029 | 0.0475 | 8.10 | 291 | 0.9507 | 0.125 | 0.0568 | 0.0536 | 6.49 | 203 | 0.2912 | 0.136 |
| HVLT Delayed Recall | 0.1499 | 0.3677 | 7.07 | 291 | 0.6839 | 0.109 | 0.2303 | 0.4096 | 6.46 | 203 | 0.5746 | 0.136 |
| HVLT Recognition | 1.4763 | 2.1106 | 4.45 | 291 | 0.4848 | 0.065 | -0.1437 | 2.4336 | 2.17 | 203 | 0.9530 | 0.033 |
| JoLO | -0.1338 | 0.1426 | 5.04 | 291 | 0.3492 | 0.076 | -0.1822 | 0.1743 | 3.43 | 203 | 0.2970 | 0.065 |
| LNS | -0.0141 | 0.0123 | 3.58 | 291 | 0.2524 | 0.050 | -0.0219 | 0.0139 | 3.63 | 203 | 0.1175 | 0.070 |
| Semantic Fluency Test | -0.0773 | 0.0490 | 1.50 | 291 | 0.1160 | 0.010 | 0.0205 | 0.0526 | 0.37 | 203 | 0.6978 | -0.019 |
| SDMT | -0.0260 | 0.0476 | 2.80 | 291 | 0.5858 | 0.036 | -0.0794 | 0.0447 | 2.70 | 203 | 0.0771 | 0.046 |

The models were adjusted for age, sex, educational levels, *APOE ε4* status and disease duration.

**Supplementary Table 3. Baseline associations between eGFR and cognitive indicators in patients with *de novo* PD stratified by sex.**

| **Cognitive indicators** | Male (n=317) | | | | | | Female (n=191) | | | | | |
| --- | --- | --- | --- | --- | --- | --- | --- | --- | --- | --- | --- | --- |
|  | β | SE | F | df | P-value | R^2^ | β | SE | F | df | P-value | R^2^ |
| CSF Aβ42 | **0.0039** | **0.0018** | **3.49** | **311** | **0.0295** | **0.038** | 0.0023 | 0.0022 | 3.28 | 185 | 0.2949 | 0.056 |
| CSF T-tau | 0.0023 | 0.0014 | 4.47 | 303 | 0.0900 | 0.053 | -0.0001 | 0.0017 | 5.17 | 184 | 0.9483 | 0.100 |
| CSF P-tau | **0.0008** | **0.0004** | **5.58** | **288** | **0.0354** | **0.072** | -0.0002 | 0.0005 | 3.67 | 174 | 0.7494 | 0.069 |
| CSF α-syn | **0.0038** | **0.0019** | **2.07** | **258** | **0.0497** | **0.020** | 0.0027 | 0.0023 | 2.82 | 145 | 0.2366 | 0.057 |
| Serum NfL | -0.0026 | 0.0015 | 46.60 | 265 | 0.0861 | 0.458 | -0.0030 | 0.0022 | 24.42 | 167 | 0.1732 | 0.405 |
| MoCA | -2167.0 | 5432.0 | 4.56 | 311 | 0.6902 | 0.053 | -6567.4 | 7116.8 | 6.44 | 185 | 0.3573 | 0.125 |
| HVLT Total Recall | 0.0460 | 0.0458 | 7.75 | 311 | 0.3156 | 0.096 | 0.0147 | 0.0567 | 7.36 | 185 | 0.7959 | 0.143 |
| HVLT Delayed Recall | 0.3951 | 0.3472 | 5.14 | 311 | 0.2560 | 0.061 | 0.0669 | 0.4305 | 9.55 | 185 | 0.8767 | 0.184 |
| HVLT Recognition | 1.0880 | 1.9590 | 3.37 | 311 | 0.5789 | 0.036 | 1.2330 | 2.5770 | 5.87 | 185 | 0.6329 | 0.114 |
| JoLO | -0.1879 | 0.1422 | 1.92 | 311 | 0.1873 | 0.014 | -0.1092 | 0.1722 | 2.64 | 185 | 0.5267 | 0.041 |
| LNS | -0.0146 | 0.0117 | 3.52 | 311 | 0.2127 | 0.039 | -0.0163 | 0.0148 | 5.56 | 185 | 0.2716 | 0.109 |
| Semantic Fluency Test | -0.0446 | 0.0464 | 0.97 | 311 | 0.3370 | 0.435 | -0.0337 | 0.0562 | 0.51 | 185 | 0.5490 | -0.013 |
| SDMT | -0.0779 | 0.0417 | 1.49 | 311 | 0.0629 | 0.008 | 0.0238 | 0.0543 | 3.09 | 185 | 0.6622 | -0.002 |

The models were adjusted for age, sex, educational levels, *APOE ε4* status and disease duration.

**Supplementary Table 4. Baseline associations between eGFR and cognitive indicators in patients with *de novo* PD stratified by *APOE ε4* status.**

| **Cognitive indicators** | *APOE ε4* (-) (n=386) | | | | | | *APOE ε4* (+) (n=122) | | | | | |
| --- | --- | --- | --- | --- | --- | --- | --- | --- | --- | --- | --- | --- |
|  | β | SE | F | df | P-value | R^2^ | β | SE | F | df | P-value | R^2^ |
| CSF Aβ42 | **0.0050** | **0.0016** | **2.76** | **380** | **0.0022** | **0.022** | -0.0009 | 0.0027 | 0.71 | 115 | 0.7368 | -0.015 |
| CSF T-tau | 0.0023 | 0.0012 | 6.83 | 376 | 0.0603 | 0.071 | -0.0009 | 0.0022 | 2.62 | 110 | 0.6781 | 0.077 |
| CSF P-tau | **0.0007** | **0.0003** | **6.52** | **359** | **0.0453** | **0.070** | -0.0002 | 0.0006 | 2.12 | 102 | 0.7743 | 0.059 |
| CSF α-syn | **0.0048** | **0.0017** | **3.91** | **303** | **0.0047** | **0.045** | -0.0001 | 0.0030 | 1.18 | 99 | 0.9793 | 0.010 |
| Serum NfL | **-0.0042** | **0.0015** | **48.14** | **332** | **0.0048** | **0.412** | -0.0002 | 0.0024 | 18.58 | 99 | 0.9340 | 0.501 |
| MoCA | -6907.0 | 5024.0 | 5.83 | 380 | 0.1700 | 0.059 | 2525.0 | 8798.0 | 3.24 | 115 | 0.7747 | 0.100 |
| HVLT Total Recall | 0.0461 | 0.0403 | 12.54 | 380 | 0.2537 | 0.130 | -0.0001 | 0.0747 | 3.98 | 115 | 0.9987 | 0.129 |
| HVLT Delayed Recall | 0.1955 | 0.3189 | 10.10 | 380 | 0.5401 | 0.106 | 0.2645 | 0.5200 | 4.64 | 115 | 0.6119 | 0.153 |
| HVLT Recognition | 0.9432 | 1.8586 | 3.99 | 380 | 0.6121 | 0.037 | 0.3944 | 2.8531 | 6.14 | 115 | 0.8903 | 0.203 |
| JoLO | -0.2185 | 0.1260 | 7.64 | 380 | 0.0837 | 0.079 | 0.0184 | 0.2236 | 3.07 | 115 | 0.9344 | 0.093 |
| LNS | -0.0178 | 0.0106 | 5.05 | 380 | 0.0929 | 0.050 | -0.0086 | 0.0186 | 2.91 | 115 | 0.6442 | 0.088 |
| Semantic Fluency Test | -0.0470 | 0.0426 | 1.13 | 380 | 0.2720 | 0.002 | -0.0115 | 0.0637 | 0.32 | 115 | 0.8570 | -0.035 |
| SDMT | -0.0399 | 0.0383 | 2.29 | 380 | 0.2980 | 0.017 | -0.0880 | 0.0696 | 1.06 | 115 | 0.2083 | 0.003 |

The models were adjusted for age, sex, educational levels, *APOE ε4* status and disease duration.

**Supplementary Table 5. Longitudinal associations between eGFR and cognitive indicators in patients with *de novo* PD stratified by age.**

| **Cognitive indicators** | Mid-age (<65 years) (n=284) | | | | Late-age (≥65 years) (n=202) | | | |
| --- | --- | --- | --- | --- | --- | --- | --- | --- |
|  | β | SE | df | P-value | β | SE | df | P-value |
| CSF Aβ42 (n=426) | -0.00206 | 0.00369 | 98.6 | 0.5778 | -0.00215 | 0.00255 | 202.0 | 0.3994 |
| CSF T-tau (n=418) | -0.00001 | 0.00004 | 182.3 | 0.7467 | **-0.00014** | **0.00005** | **82.2** | **0.0049** |
| CSF P-tau (n=395) | -0.00001 | 0.00003 | 170.4 | 0.9062 | **-0.00011** | **0.00004** | **74.9** | **0.0065** |
| CSF α-syn (n=341) | 0.00085 | 0.00110 | 65.6 | 0.4385 | -0.00017 | 0.00071 | 153.5 | 0.8110 |
| Serum NfL (n=442) | -0.00016 | 0.00028 | 231.2 | 0.5560 | **-0.00101** | **0.00031** | **81.7** | **0.0015** |
| MoCA | 155.800 | 199.600 | 218.4 | 0.4360 | 409.100 | 360.800 | 130.9 | 0.2589 |
| HVLT Total Recall | 0.00464 | 0.01904 | 247.2 | 0.8076 | 0.02438 | 0.02055 | 105.5 | 0.2381 |
| HVLT Delayed Recall | 0.05310 | 0.08032 | 257.0 | 0.5091 | 0.16210 | 0.09327 | 93.9 | 0.0855 |
| HVLT Recognition | 2.72500 | 2.07100 | 245.3 | 0.1895 | 0.47850 | 2.62280 | 130.1 | 0.8555 |
| JoLO | 0.00007 | 0.01482 | 232.9 | 0.9961 | 0.01752 | 0.01839 | 123.8 | 0.3426 |
| LNS | 0.00526 | 0.00454 | 243.5 | 0.2482 | -0.00430 | 0.00547 | 107.7 | 0.4328 |
| Semantic Fluency Test | 0.02552 | 0.01350 | 242.0 | 0.0600 | 0.01073 | 0.01775 | 116.0 | 0.5468 |
| SDMT | 0.00285 | 0.00794 | 232.0 | 0.7196 | -0.00254 | 0.00878 | 123.8 | 0.7730 |

The models were adjusted for age, sex, educational levels, *APOE ε4* status and disease duration.

**Supplementary Table 6. Longitudinal associations between eGFR and cognitive indicators in patients with *de novo* PD stratified by sex.**

| **Cognitive indicators** | Male (n=301) | | | | Female (n=185) | | | |
| --- | --- | --- | --- | --- | --- | --- | --- | --- |
|  | β | SE | df | P-value | β | SE | df | P-value |
| CSF Aβ42 (n=426) | -0.00279 | 0.00242 | 163.47 | 0.2500 | 0.00020 | 0.00348 | 120.9 | 0.9550 |
| CSF T-tau (n=418) | **-0.00010** | **0.00003** | **197.1** | **0.0075** | **-0.00004** | **0.00006** | **76.1** | **0.0075** |
| CSF P-tau (n=395) | **-0.00010** | **0.00004** | **165.2** | **0.0118** | -0.00004 | 0.00005 | 109.1 | 0.3647 |
| CSF α-syn (n=341) | -0.00028 | 0.00075 | 171.2 | 0.7064 | -0.00009 | 0.00098 | 161.8 | 0.9310 |
| Serum NfL (n=442) | **-0.00066** | **0.00026** | **200.8** | **0.0118** | -0.00021 | 0.00031 | 123.6 | 0.5059 |
| MoCA | 400.900 | 223.500 | 206.2 | 0.0743 | 557.520 | 287.360 | 95.4 | 0.0553 |
| HVLT Total Recall | **0.03795** | **0.01762** | **220.9** | **0.0323** | 0.01339 | 0.02340 | 127.1 | 0.5682 |
| HVLT Delayed Recall | **0.15517** | **0.07701** | **216.3** | **0.0452** | 0.14166 | 0.09417 | 127.2 | 0.1350 |
| HVLT Recognition | **4.19100** | **1.94700** | **239.7** | **0.0323** | -2.04600 | 2.52500 | 116.6 | 0.4194 |
| JoLO | 0.01338 | 0.01393 | 217.1 | 0.3378 | -0.01070 | 0.01801 | 131.9 | 0.5540 |
| LNS | 0.00091 | 0.00447 | 216.1 | 0.8392 | 0.00264 | 0.00478 | 133.7 | 0.5809 |
| Semantic Fluency Test | **0.03310** | **0.01314** | **224.7** | **0.0125** | 0.01964 | 0.01650 | 113.7 | 0.2360 |
| SDMT | **0.02072** | **0.00723** | **226.8** | **0.0046** | 0.00170 | 0.01111 | 129.2 | 0.8788 |

The models were adjusted for age, sex, educational levels, *APOE ε4* status and disease duration.

**Supplementary Table 7. Longitudinal associations between eGFR and cognitive indicators in patients with *de novo* PD stratified by *APOE ε4* status.**

| **Cognitive indicators** | *APOE ε4* (-) (n=371) | | | | *APOE ε4* (+) (n=115) | | | |
| --- | --- | --- | --- | --- | --- | --- | --- | --- |
|  | β | SE | df | P-value | β | SE | df | P-value |
| CSF Aβ42 (n=426) | -0.00405 | 0.00228 | 218.65 | 0.0768 | 0.00570 | 0.00394 | 68.4 | 0.1520 |
| CSF T-tau (n=418) | -0.00006 | 0.00003 | 162.3 | 0.0550 | -0.00005 | 0.00004 | 92.3 | 0.2457 |
| CSF P-tau (n=395) | **-0.00007** | **0.00003** | **182.9** | **0.0133** | -0.00002 | 0.00005 | 72.3 | 0.6904 |
| CSF α-syn (n=341) | -0.00093 | 0.00048 | 694.6 | 0.0537 | 0.00016 | 0.00057 | 238.5 | 0.7700 |
| Serum NfL (n=442) | -0.00042 | 0.00023 | 243.4 | 0.0731 | -0.00062 | 0.00039 | 838.5 | 0.1134 |
| MoCA | **405.200** | **200.200** | **244.7** | **0.0441** | **730.580** | **363.400** | **63.4** | **0.0486** |
| HVLT Total Recall | 0.01696 | 0.01594 | 256.8 | 0.2883 | **0.07365** | **0.02878** | **90.1** | **0.0121** |
| HVLT Delayed Recall | 0.10160 | 0.06700 | 264.5 | 0.1307 | **0.27800** | **0.12900** | **82.3** | **0.0341** |
| HVLT Recognition | 0.20900 | 1.74600 | 285.3 | 0.9048 | **8.13600** | **3.31200** | **75.2** | **0.0163** |
| JoLO | 0.00158 | 0.01247 | 274.5 | 0.8992 | 0.02934 | 0.02401 | 78.6 | 0.2254 |
| LNS | -0.00106 | 0.00357 | 259.4 | 0.7670 | 0.01540 | 0.00815 | 87.6 | 0.0612 |
| Semantic Fluency Test | **0.02477** | **0.01132** | **243.1** | **0.0296** | **0.05203** | **0.02344** | **94.7** | **0.0288** |
| SDMT | 0.00927 | 0.00664 | 268.3 | 0.1642 | 0.02797 | 0.01428 | 86.5 | 0.0533 |

The models were adjusted for age, sex, educational levels, *APOE ε4* status and disease duration.
